# Ultraviolet Light-Induced Surface Changes of Tungsten Oxide in Air: Combined Scanning Transmission Electron Microscopy and X-ray Photoelectron Spectroscopy Analysis

**DOI:** 10.3390/nano14181486

**Published:** 2024-09-13

**Authors:** Yuki Nakagawa, Yasuhiro Shiratsuchi, Tamaki Shibayama, Masaki Takeguchi

**Affiliations:** 1 Faculty of Engineering, Hokkaido University, N-13, W-8, Sapporo 060-8628, Japan; 2 Graduate School of Engineering, Hokkaido University, N-13, W-8, Sapporo 060-8628, Japan; 3 National Institute for Materials Science, 1-2-1 Sengen, Tsukuba 305-0047, Japan

**Keywords:** tungsten oxide, photocatalysis, identical-location scanning transmission electron microscopy, X-ray photoelectron spectroscopy, ultraviolet light, hydrocarbon decomposition

## Abstract

Scanning transmission electron microscopy (STEM) and X-ray photoelectron spectroscopy analyses were combined to clarify the ultraviolet light-induced surface changes of WO_3_ in air. Identical-location STEM (IL-STEM) analysis showed that the WO_3_ particle surface was covered with an amorphous thin film after ultraviolet irradiation in air. X-ray photoelectron spectroscopy analysis showed that hydrocarbon decomposition and the formation of carboxyl/hydroxyl species occurred. These results suggested that the amorphous thin films consisted of photocatalytic oxidative species of hydrocarbon. The IL-STEM analysis could detect small light-induced changes. This technique will be useful for the microscopic characterization of photocatalysis or photoinduced hydrophilic conversion.

## 1. Introduction

Oxide materials, such as titanium oxide (TiO_2_), tungsten oxide (WO_3_) and zinc oxide (ZnO), are widely applied in photocatalysis [1] and sensors [2,3]. Various strategies for improving the photocatalytic activity have been used for these oxide photocatalysts, including introducing defects [4,5,6], supporting cocatalysts [7,8], and fabricating heterojunctions [9,10]. In order to reveal the correlations between photocatalytic properties and these strategies, transmission electron microscope (TEM) analysis is required. Some studies have focused on the microscopic analysis of light-induced phenomena [11]. Using irradiation with ultraviolet (UV) light at 360 nm in the TEM, in situ high-resolution TEM observation has been performed for the photodecomposition of hydrocarbons on TiO_2_ films [12]. Additionally, water vapor and environmental TEM have been used for the in situ observation of the reduction of Cu_2_O to Cu under irradiation at 405 nm [13]. A laser-equipped high-voltage electron microscope has been used for the in situ observation of the photocorrosion of ZnO crystals in an ionic liquid under 325 nm irradiation [14].

The photoinduced hydrophilic conversion of a photocatalyst surface can be caused by light irradiation in air. For example, when a TiO_2_ thin film is exposed to UV light, the water contact angle (CA) decreases over time and finally reaches almost 0° to give a highly hydrophilic state [15]. The characteristics of the highly hydrophilic state have been investigated in previous studies. When the photocatalytic oxidation and photoinduced hydrophilicity of TiO_2_ and SrTiO_3_ films were investigated, both films showed almost the same photocatalytic oxidation activity but a photoinduced highly hydrophilic state was only achieved for TiO_2_ [16]. The relationship between the amount of carbon contamination and CA was investigated using X-ray photoelectron spectroscopy (XPS) and CA measurements. The highly hydrophilic state was obtained even if contaminants remained on the surface of the TiO_2_ film [17]. When the stability of the highly hydrophilic TiO_2_ surface was investigated, ultrasonication in water increased the CA to approximately 10° [18] and wet rubbing increased the CA from 3° to 80° [19]. Thus, stability can be lost with external stimuli. The removal of contaminants by oxidation can make the surface moderately hydrophilic and it appears that the highly hydrophilic state is a metastable state [15]. Structural changes on TiO_2_ surfaces under UV light have been investigated and this metastable state can be explained by an increase in the number of hydroxyl (OH) groups on the TiO_2_ surface [15]. The XPS O 1s spectrum for highly hydrophilic TiO_2_ surfaces shows a broad shoulder peak to the higher binding energy side of the lattice oxygen main peak. This shoulder is fitted by two species of hydroxyl groups and physically adsorbed molecular water, and gradually decreases during storage in the dark [17]. The hydrophilic conversion of a WO_3_ thin film has been also reported in a previous study, where the CA decreased to below 5° under UV or visible light irradiation in air. It has been suggested that the presence of oxygen and water molecules is important in photoinduced hydrophilic conversion [20]. Additionally, a study has shown that photogenerated holes are required to achieve highly hydrophilic conversion [20]. In a cocatalyst system with WO_3_ and Pt, photoinduced hydrophilic conversion occurred for WO_3_ films with underlying Pt nanoparticles, but the hydrophilicity decreased for films with overlying Pt nanoparticles because of the hydrophobic nature of the Pt nanoparticles [21]. In WO_3_ films fabricated using a W^6+^ complex salt of citric acid, visible light-induced hydrophilicity was achieved and the interaction between water molecules and the oxygen-deficient WO_3_ thin films was important for achieving hydrophilic conversion [22].

In the present study, to clarify the UV-induced structural changes of WO_3_ in air, we performed an atomic-level observation of WO_3_ using a spherical aberration-corrected scanning transmission electron microscope (*Cs*-corrected STEM). The WO_3_ nanoparticles were analyzed by identical-location (IL) STEM analysis. In one previous study, oxygen vacancy-induced edge reconstruction was observed at the atomic scale in a high-angle annular dark-field (HAADF) STEM image [23]. In this study, an ozone lamp was used as the UV light source. In another study, the ozone lamp irradiation of WO_3_ photoanodes in air yielded a higher surface area for WO_3_, but the process for this was not clarified [24]. Slight structural changes on the surface were observed by IL-STEM after the UV irradiation. We also performed XPS analysis before and after UV irradiation.

## 2. Materials and Methods

### 2.1. Identical-Location-STEM (IL-STEM) Analysis

WO_3_ nanopowders (particle size: <100 nm, purity: N.A., Sigma–Aldrich Japan G.K., Tokyo, Japan) were used in this analysis. X-ray diffraction (XRD) measurements were performed using a diffractometer with Cu Kα radiation (MiniFlex, Rigaku, Tokyo, Japan). The WO_3_ particles were dispersed in ethanol and a few drops of the suspension were placed on a microgrid with a carbon-supporting membrane and dried in air. The samples were observed using *Cs*-corrected STEM (JEM-ARM200F, JEOL) with a cold field emission gun operated at an acceleration voltage of 200 kV. First, HAADF-STEM images were obtained for some regions of the particles. Then, the TEM grid with WO_3_ powder was taken out of the TEM and UV light irradiation was performed in air for 4 h. An ozone lamp (GL-4Z, electric power: 4 W, main wavelengths: 254 nm and 185 nm, Kyokko Denki, Tokyo, Japan) was used for the UV irradiation. The distance between the lamp and sample was approximately 1 cm. The temperature and the humidity during the irradiation were approximately 25 °C and 15%, respectively. To remove ozone species, the irradiation experiment was conducted in a fume hood. After UV irradiation for 4 h, the identical-location of the sample was again observed by STEM. An atomic structure model of the WO_3_ was prepared using ReciPro software (Ver. 4.876).

### 2.2. XPS Analysis

Tungsten plates (purity: 99.95%, 10 mm × 10 mm × 0.2 mm, Nilaco, Tokyo, Japan) were calcined in air at 800 °C for 0.5 h to synthesize WO_3_ plates. UV irradiation was performed in air using the same ozone lamp as in Section 2.1. The distance between the lamp and sample was approximately 1 cm. After UV irradiation, the prepared plates were stored in a vacuum desiccator. After storage, the plates were analyzed by XPS (JPS-9200, Mg Kα radiation, JEOL, Tokyo, Japan). First, we prepared a pristine WO_3_ plate (A-1) and a plate irradiated for 12 h (B-1) for XPS analysis (Table 1). The temperature and humidity in the room were recorded using a digital thermo-hygrometer (PC-7980GTI, Sato Keiryoki Mfg, Tokyo, Japan) before and after the light irradiation experiments. For the B-1 sample, the temperature and the humidity during the experiment were approximately 17.9 °C and 30%, respectively. We then irradiated the A-1 and B-1 plates again under humid conditions, and these plates were labeled as A-2 and B-2, respectively. The A-2 and B-2 plates were analyzed by XPS 1 day after the UV irradiation (Table 1).

The chemical bonding states in the prepared plates were analyzed by XPS. The base pressure during spectra acquisition was better than 5 × 10^−6^ Pa, which was achieved using a turbomolecular pump. The area of sample analyzed was 3 mm in diameter. Neither sputter cleaning nor a charge neutralizer was used. The work function method was used for the calibration of the binding energy values [25]. The work function of WO_3_ is reportedly 5.05 eV [26]; thus, the binding energy of the C 1s peak was calibrated at 284.53 eV in this work. The background was subtracted using Shirley’s method. Peak deconvolution and quantification were carried out using SpecSurf software (Ver. 1.9.4.3). A mixed Gaussian–Lorentzian function was used for peak deconvolution. The atomic ratios of C, O, and W were derived from the integrated intensities of the C 1s, O 1s, and W 4f peaks. The relative sensitivity factors of C 1s, O 1s and W 4f_7/2_ in the SpecSurf software were used for the calculation.

## 3. Results and Discussion

**Figure 1:**
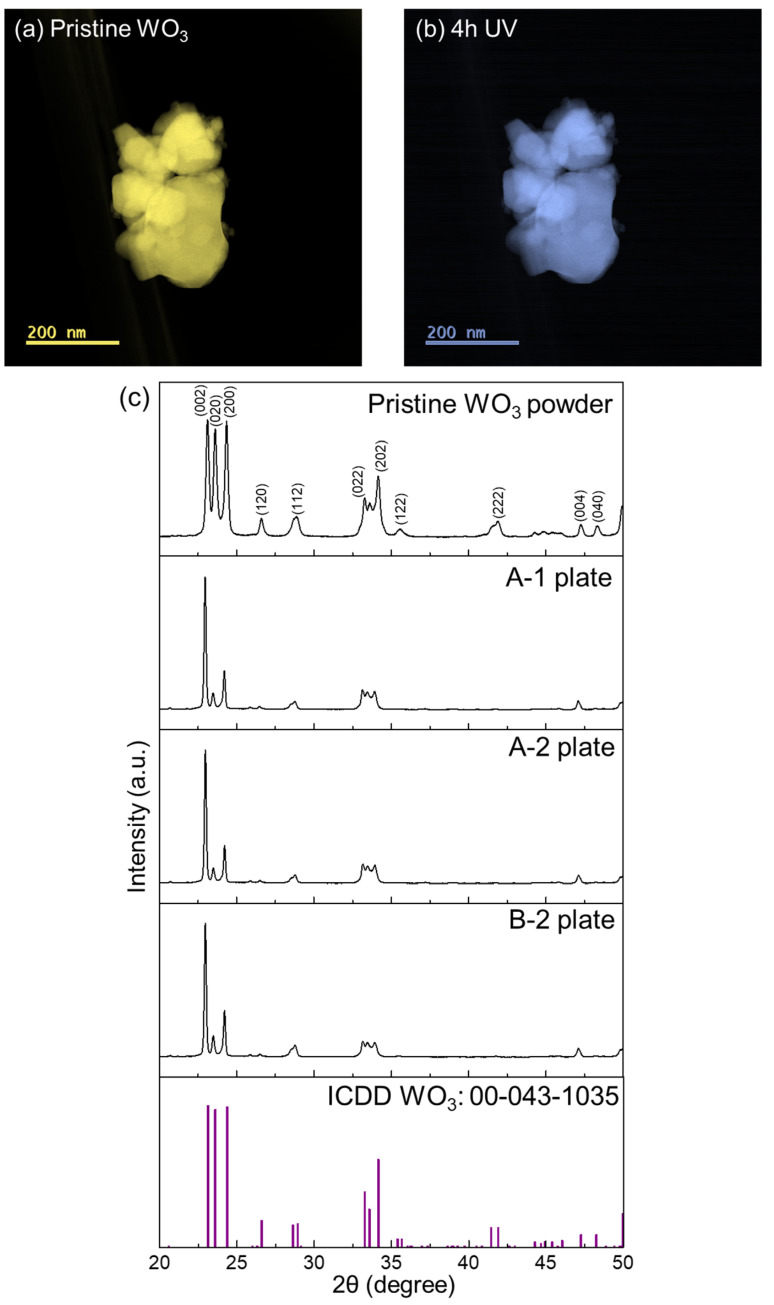
(**a**,**b**) HAADF-STEM images of WO_3_ powders: (**a**) pristine WO_3_, and (**b**) after 4 h of UV irradiation. (**c**) XRD profile the WO_3_ powder, plates (A-1, A-2, and B-2), and reference pattern of monoclinic WO_3_.

**Figure 2:**
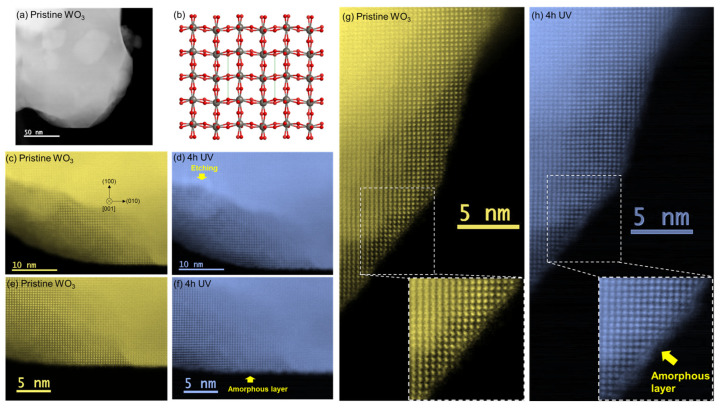
(**a**) High-magnification HAADF-STEM image of pristine WO_3_ particles. (**b**) Atomic structure model of monoclinic WO_3_ along the [001] zone axis. Gray atoms and red atoms represent W and O, respectively. (**c**–**h**) Atomic-scale HAADF-STEM images of WO_3_ particles before and after UV irradiation.

**Figure 3:**
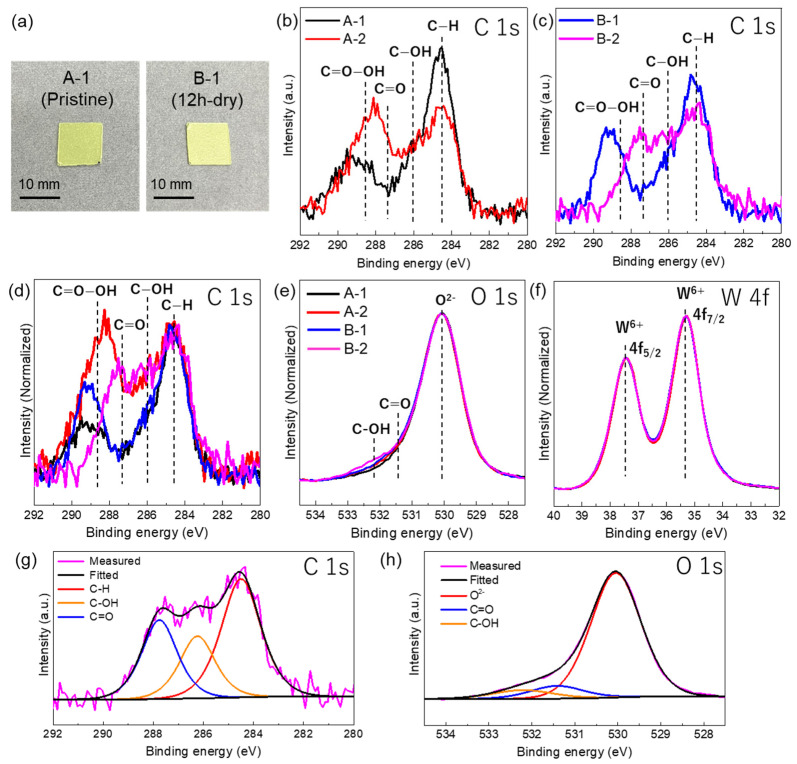
(**a**) Photographs of the A-1 and B-1 plates. (**b**) C 1s XPS spectra of the A-1 and A-2 plates. (**c**) C 1s XPS spectra of the B-1 and B-2 plates. (**d**) Normalized C 1s XPS spectra of all the plates. Explanation of each color line was same for that of Figure 3e. (**e**) Normalized O 1s XPS spectra of all the plates. (**f**) Normalized W 4f XPS spectra of all the plates. Explanation of each color line was same for that of Figure 3e. (**g**) C 1s spectra of the B-2 plate deconvoluted into three peaks. (**h**) O 1s spectra of the B-2 plate deconvoluted into three peaks.

At the magnification used in HAADF-STEM, no change was observed in the morphology of the WO_3_ particles before and after UV light irradiation (Figure 1a,b). For the pristine WO_3_ powder, the diffraction peaks in the XRD profile (Figure 1c) were indexed to a monoclinic WO_3_ (γ-WO_3_, ICDD No. 00-043-1035). In Figure 1c, the XRD profiles of plate samples are also shown, and the results are discussed in the next section. Atomic-scale HAADF-STEM images of the particle edges were obtained (Figure 2). A HAADF image of the pristine WO_3_ particles was obtained at high magnification (Figure 2a). An atomic structure model of monoclinic WO_3_ along the [001] zone axis showed overlapping W-O atomic columns and oxygen atomic columns (Figure 2b). Atomic-scale HAADF-STEM images of the WO_3_ particles were observed along the [001] zone axis (Figure 2c–h). Because HAADF-STEM imaging is incoherent, and the image contrast is roughly proportional to Z^1.6–2.0^ (Z: atomic number), the bright dots in Figure 2c–h corresponded to overlapping W-O columns. In the atomic-scale image, some slight changes were observed after UV irradiation in air. We performed IL-STEM analysis immediately after the 4 h UV irradiation. Morphological changes caused by etching were observed in one region (arrow in Figure 2d). Large quantities of carbon species could exist in this region, and this was confirmed by XPS measurements, which showed that carbon contamination decreased after UV irradiation (Figure 3b–d and Table 2). The amorphous layer covering the particle edge became thicker (Figure 2e,g compared with Figure 2f,h). The arrangement of overlapping W-O columns in the particles was similar before and after UV irradiation, which suggested that the internal crystal structure was not changed by the UV light. However, the particle surface was covered by an amorphous layer after UV irradiation in air. The UV light-induced structural changes of WO_3_ in air were characterized by IL-STEM. This analysis minimized the effect of electron irradiation during the observation. Slight structural changes induced by UV light would be difficult to detect using only ex-situ STEM analysis in different locations. Information about the amorphous layer after UV irradiation is provided later in this section.

The XRD profiles of the plate sample (A-1, A-2, B-2) are shown in Figure 1c. The diffraction peaks of all the profiles were indexed to monoclinic WO_3_. A different peak intensity ratio in the plate samples compared with powder would originate from the preferred orientation of plates or the coexistence of oxygen deficient phase [5]. The XRD profiles of A-2 and B-2 were similar compared with that of A-1, indicating that the internal crystal structures were not changed by UV irradiation. XPS analysis of the WO_3_ plates was used to study the surface chemical bonding states before and after UV light irradiation. The pristine WO_3_ plate (A-1) and the plate after 12 h of irradiation (B-1) were both yellow (Figure 3a), which is a typical color for stoichiometric WO_3_ [5]. The A-2 and B-2 plates were also yellow. If the non-stoichiometric oxygen-deficient WO_3-x_ phase formed, the color would change to blue [5]. The XPS C 1s spectra (Figure 3b–d) showed peaks for C–H (284.5 eV), C–OH (286.0 eV), C=O (287.3 eV), and C=O–OH (288.7 eV) [27]. After the 4 h irradiation (plate A-2), the C–H peak intensity decreased and the C=O–OH peak of plate A-1 shifted toward the C=O peak (Figure 3b). The XPS C 1s spectrum of the B-1 plate (Figure 3c) showed peaks for C–H and C=O–OH. In the XPS spectrum of plate B-2 (Figure 3c), the C–H peak intensity decreased and a broad peak for C=O and C–OH was observed. The XPS C 1s spectra of all samples were compared by normalizing the spectra to the C–H peak (Figure 3d). The C–H and C=O–OH peaks were observed for the A-1 and B-1 plates. The intensity of the C–H and C=O–OH peak was decreased, and a peak at a lower binding energy (C=O peak) appeared for A-2 and B-2. In the normalized O 1s spectra (Figure 3e), a peak corresponding to the lattice oxygen of WO_3_ was observed at 530.0 eV [5]. Compared with pristine WO_3_, the plates with UV irradiation showed slight increases in the peak intensities for C=O (531.4 eV) and C–OH (532.2 eV) [28,29]. Plate B-2 (long irradiation period) had the strongest peaks. In the normalized W 4f spectra (Figure 3f), all the spectra overlapped. W 4f_5/2_ and W 4f_7/2_ spin-orbit doublet peaks corresponding to W^6+^ were observed at 37.4 eV and 35.3 eV, respectively [5]. These results indicated that the W was in the form of WO_3_ for all samples. In the peak fitted C 1s spectra of the B-2 plate (Figure 3g), peaks were observed for C–H, C–OH, and C=O. For the peak fitted O 1s spectra of the B-2 plate (Figure 3h), although the contribution of the lattice oxygen of WO_3_ was strong, C=O and C–OH peaks were also observed. The results from the XPS spectra were used for quantification (Table 2). For the A-1 and A-2 plates, the C/W and O/W ratios were slightly different. For the B-2 plate compared with the B-1 plate, the C/W ratio decreased and the O/W ratio was increased. Thus, the long period of UV irradiation decreased the amount of carbon and increased the amount of oxygen.

It is known that the contamination of hydrocarbon is widely used for charge correction in XPS analysis. The most likely source of hydrocarbon is the air atmosphere. A recent study investigated the source of contaminations and suggested that the accumulation of volatile organic compounds in air form contaminations on the surface of materials [30]. In this study, the existence of contaminations would have also been caused by air exposure. The changes observed in the XPS spectra with UV irradiation (Figure 3) were similar to those observed for a TiO_2_ film system in a previous study [31]. In this earlier study, UV irradiation in an oxygen atmosphere was performed for both hydrophilic and hydrophobic TiO_2_ films. The C 1s spectrum of the hydrophilic film showed that large amounts of hydrocarbon were removed, and the O 1s spectra for both films showed that hydroxide groups were adsorbed [31]. This suggests that the hydrocarbon decomposition observed in the present study (Figure 3b–d) was caused by photocatalytic oxidation on the WO_3_ surface. As shown in the XPS spectra of the A-1 and B-1 samples, carboxyl (C=O–OH) species were detected in the first period of decomposition. The formation of carboxyl species was also reported for the TiO_2_ photocatalyst system [32]. For the photodegradation of the acetaldehyde system, the amount of CO_2_ produced by acetaldehyde decomposition was low because stable intermediates, such as acetic acid and formic acid, accumulated on the WO_3_ surface [33]. Thus, the low photocatalytic activity of pristine WO_3_ would result in the existence of carboxyl species in the XPS spectra even with the longer UV irradiation period (plate B-1). The humidity during UV irradiation is also important. A comparison of the C 1s spectra of A-1 and A-2 showed that the peak for C=O–OH species shifted to a lower binding energy for A-2. Although the C=O–OH peak was present for the B-1 plate, its intensity was low for the B-2 plate and peaks were observed for C=O and C–OH species. These results showed that the photocatalytic decomposition of C=O–OH species occurred under humid conditions. The total amount of carbon in A-2 was similar to that in A-1, but the amount in B-2 was significantly lower (Table 2); thus, the full decomposition of hydrocarbons would be slow for pristine WO_3_. The surface of the particle was covered with an amorphous layer after the UV light irradiation (Figure 2). Considering the UV irradiation conditions in the IL-STEM analysis (4 h under dry conditions) and the XPS results, the amorphous layer could be formed by intermediates containing carboxyl groups that are produced by the photocatalytic oxidation of hydrocarbon species. In future, IL-STEM could be combined with electron energy loss spectroscopy to analyze photocatalysts and clarify their site-dependent photocatalytic decomposition behaviors (e.g., defect sites and noble metal cocatalyst sites).

## 4. Conclusions

UV light-induced surface changes on WO_3_ were characterized using IL-STEM and XPS analyses. The XPS analysis showed that hydrocarbon decomposition and hydroxyl species formation occurred under UV irradiation in air. For the hydrocarbon decomposition, chemical bonding states similar to carboxyl species were observed as intermediates, and then these species decomposed into C=O and C–OH. For the IL-STEM analysis, UV light-induced surface changes of WO_3_ in air were observed at the atomic scale. HAADF-STEM observation along the [001] zone axis showed that the particle surface was covered with an amorphous thin film after UV irradiation; however, the internal crystal structure did not change. Taking the XPS results into consideration, the formation of amorphous layers can be induced by the photocatalytic oxidation of hydrocarbon species by UV light. IL-STEM can detect slight morphological changes caused by light-induced reactions. This method will be useful for the atomic-scale analysis of photocatalysis or photo-induced hydrophilic conversion.

## Figures and Tables

**Table 1:** Sample treatment conditions, number of days that passed between UV irradiation and the date of XPS analysis, and average humidity and temperature used for the sample treatment.

Sample	Sample Treatment	The Number of Days That Passed between UV Irradiation and the Date of XPS Analysis	Average Humidity (%)	Average Temperature (°C)
A-1	None (pristine)	–	–	–
B-1	12 h irradiation/dry	108	30	17.9
A-2	A-1 + 4 h irradiation/humid	1	72	27.7
B-2	B-1 + 4 h irradiation-humid	1	72	27.7

**Table 2:** Quantification results (atomic percent [at%] and ratios) from XPS analysis.

Sample	Sample Treatment	C (at%)	O (at%)	W (at%)	C/W Ratio	O/W Ratio
A-1	Pristine	23.5	55.0	21.5	1.09	2.56
B-1	12 h irradiation/dry	22.8	55.8	21.4	1.07	2.61
A-2	A-1 + 4 h irradiation/humid	23.9	54.8	21.3	1.12	2.57
B-2	B-1 + 4 h irradiation/humid	19.8	58.4	21.8	0.91	2.68

## Data Availability

Data will be made available upon request.
